# Melatonin influence in ovary transplantation: systematic review

**DOI:** 10.1186/s13048-016-0245-8

**Published:** 2016-06-10

**Authors:** M. E. Shiroma, N. M. Botelho, L. L. Damous, E. C. Baracat, J. M. Soares-Jr

**Affiliations:** Disciplina de Ginecologia, Departamento de Obstetrícia e Ginecologia, Hospital das Clínicas, Faculdade de Medicina, Universidade de São Paulo, Av. Dr. Enéas de Carvalho Aguiar, 255 - Cerqueira César, São Paulo, SP 05403-000 Brazil; Disciplina de Ginecologia, Departamento de Obstetrícia e Ginecologia, Faculdade de Medicina, Universidade Federal do Pará, Av. Generalíssimo Deodoro, 01 - Umarizal, Belém, Pará 66050-160 Brazil; Laboratory of Molecular and Structural Gynecology, Faculdade de Medicina, Universidade de São Paulo, Avenida Doutor Arnaldo, 455 - Cerqueira César, São Paulo, SP 01246-904 Brazil

**Keywords:** Melatonin, Ovary, Transplant

## Abstract

Melatonin is an indolamine produced by the pineal gland and it can exert a potent antioxidant effect. Its free radical scavenger properties have been used to advantage in different organ transplants in animal experiments. Several concentrations and administration pathways have been tested and melatonin has shown encouraging beneficial results in many transplants of organs such as the liver, lungs, heart, pancreas, and kidneys. The objective of the present study was to review the scientific literature regarding the use of melatonin in ovary transplantation. A systematic review following the Preferred Reporting Items for Systematic Reviews and Meta-Analyses (PRISMA) statement was carried out using the Cochrane and Pubmed databases and employing the terms ‘melatonin’ AND ‘ovary’ AND ‘transplantation.’ After analysis, 5 articles were extracted addressing melatonin use in ovary transplants and involving 503 animals. Melatonin enhanced various graft aspects like morphology, apoptosis, immunological reaction, revascularization, oxidative stress, and survival rate. Melatonin’s antioxidative and antiapoptotic properties seemingly produce positive effects on ovarian graft activity. Despite the promising results, further studies in humans need to be conducted to consolidate its use, as ovary transplantation for fertility preservation is gradually being moved from the experimental stage to a clinical setting.

## Background

Melatonin is an indolamine produced mainly by the pineal gland [1, 2] and it has a potent freeradical scavenger activity with subsequent antioxidant and antiapoptotic functions [1, 3, 4]. Unlike vitamin C, which is hydrophilic, and vitamin E, which is lipophilic, the melatonin molecule carries both hydrophilic and lipophilic affinities and therefore diffuses broadly in diverse subcellular compartments like the membranes, cytoplasm, nucleus, and mitochondria [1, 4]. It has thus the ability to produce its antioxidative action rapidly and effectively as soon as the oxidative agents are produced [5]. There are reports about melatonin action on the rat ovary specific receptors MT1 and MT2 [6].

There are also studies indicating that one of the main challenges in achieving a successful transplant, is the reduction in graft follicular loss and damage. This is attainable through mitigation of the free radicals produced by the procedure [7] and primarily originating from ischemia-reperfusion distress [5]. Melatonin’s immunological and antiapoptotic properties are seen as potentially implicated in the enhancement of transplantation success [5], a welcome improvement even in heterologous and autologous grafting. There are other studies demonstrating the benefits of melatonin in experimental transplants of organ, like the liver [8], lungs [9], heart [10], pancreas [11, 12] and kidneys [13]. Enhancement of the graft function, immunological compliance, and antiapoptotic and antioxidative status are examples of the positive outcome.

As there is evidence that melatonin’s properties may experimentally improve the transplants of a variety of organs along different pathways, the aim of this review was to gather published studies of the indolamine’s use in ovary transplantation.

## Methods

In this systematic review, the PubMed and Cochrane databases were searched for reports published in any language between October 1, 2003 – date of the first ever published related paper [14] – and October 31, 2015, with the search terms: ‘melatonin’ AND ‘ovary’ AND ‘transplantation.’

Data were extracted from the selected articles. Quality assessment was performed independently by two reviewers (M.E.S. and N.M.B.). When there was any disagreement, a third reviewer (L.L.D.) was consulted. The analysis followed the PRISMA statement for systematic reviews [15].

**Fig. 1:**
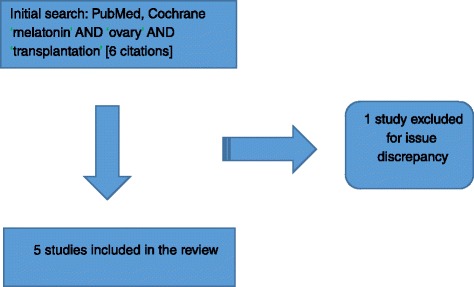
Algorithm of selected studies

Six studies were identified and after detailed analysis of the content, one of them [16] was excluded for issue discrepancy (Fig. 1).

## Results

All of the selected studies were published in the 12 years previous to this review. A total of 503 rodents (mice and rats) were studied. There are no reports of animal loss. Notwithstanding a study which used 5 human xenotransplanted ovarian samples, all were studies which experimentally analyzed the effects of melatonin on animals.

Except in the pioneer study by Sapmaz [14], who employed fresh ovaries, in all other researches, grafts were frozen and thawed. Many different concentrations (for the maintenance solution of the graft) and application routes (oral and intraperitoneal) were studied. Two studies adopted similar methodology and could be paired [17, 18].

The biological effects of melatonin on graft were enhancement of morphology; revascularization; and improved survival rate with concomitant reduction in apoptosis, immunological Th1/Th2 lymphocyte reaction, and oxidative stress (a decrease in oxidative factors and an increase in antioxidative agents). Results are summarized in Table 1.

**Table 1:** Effects of melatonin on ovary transplantation

Author	Species	Study model	Melatonin route and dosage	Results of melatonin use
Friedman, 2012 [3]	Human; Nu/nu Balb/c mice	Xenotransplanted thawed graft; donor: 5 cancer patients aged 6–23 years; recipient: 79 immunodeficient nu/nu Balb/c mice aged 10–12 weeks	Oral administration in feeding water; 240 mg/L	Reduced number of apoptosis and atretic follicles
Hemadi, 2012 [19]	Balb/c mice	Heterologous thawed graft; donor: mice aged 10 days; recipient: 180 mice aged 8–10 weeks; 900 transplants	Oral administration; 20–200 mg/kg/day	Enhanced follicle quality, quantity, and graft size with low dosage; diminished Th1/Th2 immunological reaction and longer graft lifespan with high dosage
Hemadi, 2011 [18]	F1 hybrid mice	Heterologous thawed graft; donor: mice aged 10 days; recipient: 60 mice aged 8–10 weeks	Graft: 100 μM PBF Recipient: intraperitoneal for 2 days; 20 mg/kg/day	Enhanced corpora lutea, secondary and antral follicles
Hemadi, 2009 [17]	F1 hybrid mice	Heterologous thawed graft; donor: 120 mice aged 10 days; recipient: 36 mice aged 8–10 weeks	Graft: 100 μM PBF Recipient: intraperitoneal for 2 days; 20 mg/kg/day	Improved mean graft survival, ovary size, and revascularization
Sapmaz, 2003 [14]	Wistar albino rats	Autologous fresh graft; 28 Wistar albino rats aged 12–14 weeks	intraperitoneal prior to transplantation; 20 mg/kg	Diminished ovarian and plasmatic malondialdehyde and ovarian necrosis; enhanced glutathione peroxidase and superoxide dismutase

## Conclusions

The potential effects of melatonin use in animal ovary transplantation align with previously reported positive findings of its application in many experimental transplants of such organs as the liver [8], lungs [9], heart [10], pancreas [11, 12] and kidneys [13].

In our review we found evidence of the effects of melatonin on ovarian graft. Notwithstanding the administration routes (oral or intraperitoneal) and vehicles (graft), the selected studies reported a promising performance of the indolamine. This points to a wide solubility which enables it to spread rapidly throughout a variety of tissues. The effects comprised a wide range of properties, including follicle morphology and dynamics, apoptosis, graft survival range, immunologic activity and antioxidative mechanisms. The potent antioxidative properties of melatonin, along with its free radical scavenger activity, decrease oxidative stress and thus increase the survival rate. Melatonin not only acts directly as an anti-free radical agent, but also activates other enzymes, such as superoxide dismutase, glutathione peroxidase, and catalase [20, 21], with the potential to decrease oxidative damage. Even melatonin metabolites have free radical scavenger properties, thus triggering multiple synergistic antioxidative factors, a phenomenon referred to as the cascade effect [20, 21]. The antiapoptotic effect was also remarkable and is based on reduction of Bcl2 expression and caspase-3 activity [22]. The enhancement of ovarian graft function is also attributable to melatonin, given that it is known to modulate steroidogenesis and ovulatory function [23, 24]. The subsequent balance between free radicals and antioxidative substances in the ovarian follicle also improves oocyte and granulosa cell function [1, 2, 21]. Additionally, the indol also attenuated immunological reaction and improved revascularization, two essential properties for a successful graft allocation.

The present review has some limitations imposed by the studies it covered. The heterogeneity of methods made it difficult to compare results. The wide range of apparently beneficial melatonin action on ovarian transplantation is quite encouraging, but melatonin was studied only in animal models. Moreover, except for the Sapmaz [14] study, the other studies employed heterologous or xenotransplantation, which differs from clinical preservation of human fertility, for this method requires autologous transplantation. Melatonin was administered in the feeding water in two studies [3, 19]. Therefore, different water intake by the animals might have resulted in diverse plasmatic concentration of melatonin. Apart from Hemadi [19], who dosed melatonin plasmatic levels, the authors did not determine the in vivo availability of melatonin in blood or urine. Furthermore, no study used control animals (not even pinealectomized rodents or rodents submitted to continuous light) to isolate the effect of endogenous melatonin production.

Melatonin’s antioxidative and antiapoptotic properties seem to produce positive effects on ovarian graft. Despite the promising results, further studies are needed in humans to consolidate its use, as ovary transplantation for fertility preservation is gradually being moved from the experimental stage to a clinical setting.

## Abbreviation

PRISMA, Preferred Reporting Items for Systematic Reviews and Meta-Analyses.
